# Surveying awareness of hand hygiene guidelines in the Sanin region of Japan

**DOI:** 10.1186/1753-6561-5-S6-P115

**Published:** 2011-06-29

**Authors:** T Horii, Y Yoshida, K Komatsu

**Affiliations:** 1 Infection Control Division, Tottori University Hospital, Yonago, Japan; 2 Saraya Co., Ltd., Osaka, Japan

## Introduction / objectives

Given the history of the 2002 CDC guidelines usage in Japan, we surveyed the awareness and adoption of WHO Guideline on Hand Hygiene in Health Care published in 2009, particularly the Five Moments of Hand Hygiene tool by healthcare workers (HCWs) in Japan.

## Methods

A questionnaire survey was conducted on 64 HCWs at the Tottori Prefecture Healthcare Associated Infection Control Seminar held on November, 2010.

## Results

The rates for awareness on the WHO and CDC hand hygiene guidelines were 26 (41%) and 28 (44%) of HCWs, respectively. 14 (22%) of HCWs were able to explain the WHO Five Moments of Hand Hygiene tool when asked, and 13 (20%) had seen the tool but could not explain its meaning. The subject HCWs had reported being introduced to the WHO guideline tools through publications, reviews in Japanese scholarly journals and in seminar presentations. The rest (58%)had no prior knowledge of the tool. All the subjects had never read the WHO guideline in English or a Japanese translation. However, 6 (9%) of the subjects had read the CDC guidelines. In addition, 43 (67%) of HCWs were willing to read the WHO guideline if a Japanese translation were available. There were many reasons why the survey subjects had not read the WHO Guideline, but the foremost reason was the lack of a Japanese translation.

## Conclusion

During this investigation, we found that the HCWs who were aware of the new WHO guideline identified it with the included tools such as the 5 Moments of Hand Hygiene illustration, suggesting the strong impression of visual aids for HCW workplace education. Our results showed that many Japanese HCWs were willing to consider the WHO guidelines if a Japanese translation were available, which is thought to be necessary in releasing a Japanese translation of high quality as soon as possible and fostering faster adoption throughout the country.

## Disclosure of interest

None declared.

